# From immune to olfactory expression: neofunctionalization of formyl peptide receptors

**DOI:** 10.1007/s00441-020-03393-5

**Published:** 2021-01-16

**Authors:** Madlaina Boillat, Alan Carleton, Ivan Rodriguez

**Affiliations:** 1 Department of Genetics and Evolution, Faculty of Sciences, University of Geneva, quai Ernest-Ansermet 30, 1211 Geneva, Switzerland; 2 Department of Basic Neurosciences, Faculty of Medicine, University of Geneva, 1 rue Michel-Servet, 1211 Geneva, Switzerland

## Abstract

Variations in gene expression patterns represent a powerful source of evolutionary innovation. In a rodent living about 70 million years ago, a genomic accident led an immune formyl peptide receptor (FPR) gene to hijack a vomeronasal receptor regulatory sequence. This gene shuffling event forced an immune pathogen sensor to transition into an olfactory chemoreceptor, which thus moved from sensing the internal world to probing the outside world. We here discuss the evolution of the FPR gene family, the events that led to their neofunctionalization in the vomeronasal organ and the functions of immune and vomeronasal FPRs.

The immune and olfactory systems, despite being respectively devoted to probe the internal and external worlds, face a very similar challenge: they must recognize millions of different molecules. An obvious answer to this task, which was the one selected during vertebrate evolution, is to build very large chemosensory toolboxes. The immune system generates this tool diversity using an original genomic recombination strategy, which, starting with a very limited number of genes, is able to produce millions of different transcripts. What evolution selected for the olfactory system is less parsimonious. It is in the numbers that diversity is achieved in the nose. Thus, mice, elephants, and most mammalian species benefit today from a vast repertoire of olfactory chemoreceptor genes, which in some species number over 5% of their coding genes. But the parallel between both systems does not end here. The present review points to such a parallel and will highlight a remarkable interaction between the immune and olfactory chemosensory toolboxes.

## Expression and function of FPRs in the immune system

Formyl peptide receptors (FPRs) are seven transmembrane domain G-protein coupled receptors belonging to the rhodopsin-like superfamily. They are expressed in myeloid cells, mainly in neutrophils and monocytes, but also in a number of tissues and cells including the lymphoid cells, spleen, platelets, bone marrow, hepatocytes, astrocytes, neurons, microglia, immature dendritic cells, and epithelial cells (extensively reviewed in Migeotte et al. 2006; Weiß and Kretschmer 2018; Ye et al. 2009). FPRs play an important role in host defense against pathogens. They respond to a broad range of natural ligands, including exogenous pathogen-derived compounds and endogenous molecules that signal cellular dysfunction. Immune FPRs detect molecular signatures of bacteria and other microorganisms, acting as chemotactic pattern recognition receptors (PRRs). The activation of immune FPRs triggers various antimicrobial responses, including the migration of leukocytes towards infection sites, phagocytosis, degranulation, and the release of oxidants (Ye et al. 2009). Neutrophils expressing FPRs were initially shown to be attracted by the formyl tri-peptide fMet-Leu-Phe (fMLF) (Schiffmann et al. 1975). Later, the repertoire of FPR ligands, in particular for the human FPR1, was extended to include a large number of N-formylated peptides derived from bacteria (Bufe et al. 2015; Rabiet et al. 2005; Ye et al. 2009). Bacteria (as well as mitochondria and chloroplasts) initiate translation with an *N*-formylmethionine, and the cleavage during the translocation process of the signal sequence containing this *N*-formylmethionine is a potential source of FPR ligands (Dalbey et al. 2012). These signal peptides are evolutionarily conserved among bacteria, and their repertoire has been estimated to reach up to one billion (Bufe and Zufall 2016). They thus constitute a very large pool of pathogen-associated molecular patterns (PAMPs), which potentially activate immune FPRs. As mentioned, bacteria are not the sole source of N-formylated peptides. Thus, unsurprisingly, some host-derived N-formylated peptides, produced by mitochondria, can also activate immune FPRs (Carp 1982; Rabiet et al. 2005). But the promiscuous repertoire of FPR ligands is not restricted to N-formylated peptides. Indeed, immune FPRs, more specifically the human FPR2, were shown to respond to a diverse array of ligands, including HIV-derived peptides, staphylococcal-derived phenol-soluble modulins, molecules involved in inflammatory processes such as annexin I, lipoxin A4 or the urokinase-type plasminogen activator receptor uPAR, and antimicrobial peptides such as CRAMP and amyloidogenic proteins (Weiß and Kretschmer 2018; Ye et al. 2009). Such variety of ligands, both in terms of origin and chemical characteristics, did not help in the determination of the primary function of immune FPRs. Nevertheless, in vivo studies, with mice lacking FPR1 (mFpr1) and/or FPR2 (mFpr2), have revealed the essential role of these receptors both in host defense processes against invading bacterial pathogens, as well as during inflammation and tissue damage. For example, immune FPRs mediate bacterial clearance after *Streptococcus pneumonia* or *Staphylococcus aureus* infection, the regulation of inflammatory responses during bacterial meningitis, the reduction of injury to distant organs during polymicrobial sepsis, and the promotion of wound healing induced by damage-associated molecular patterns (Weiß and Kretschmer 2018; Ye et al. 2009).

**Fig. 1:**
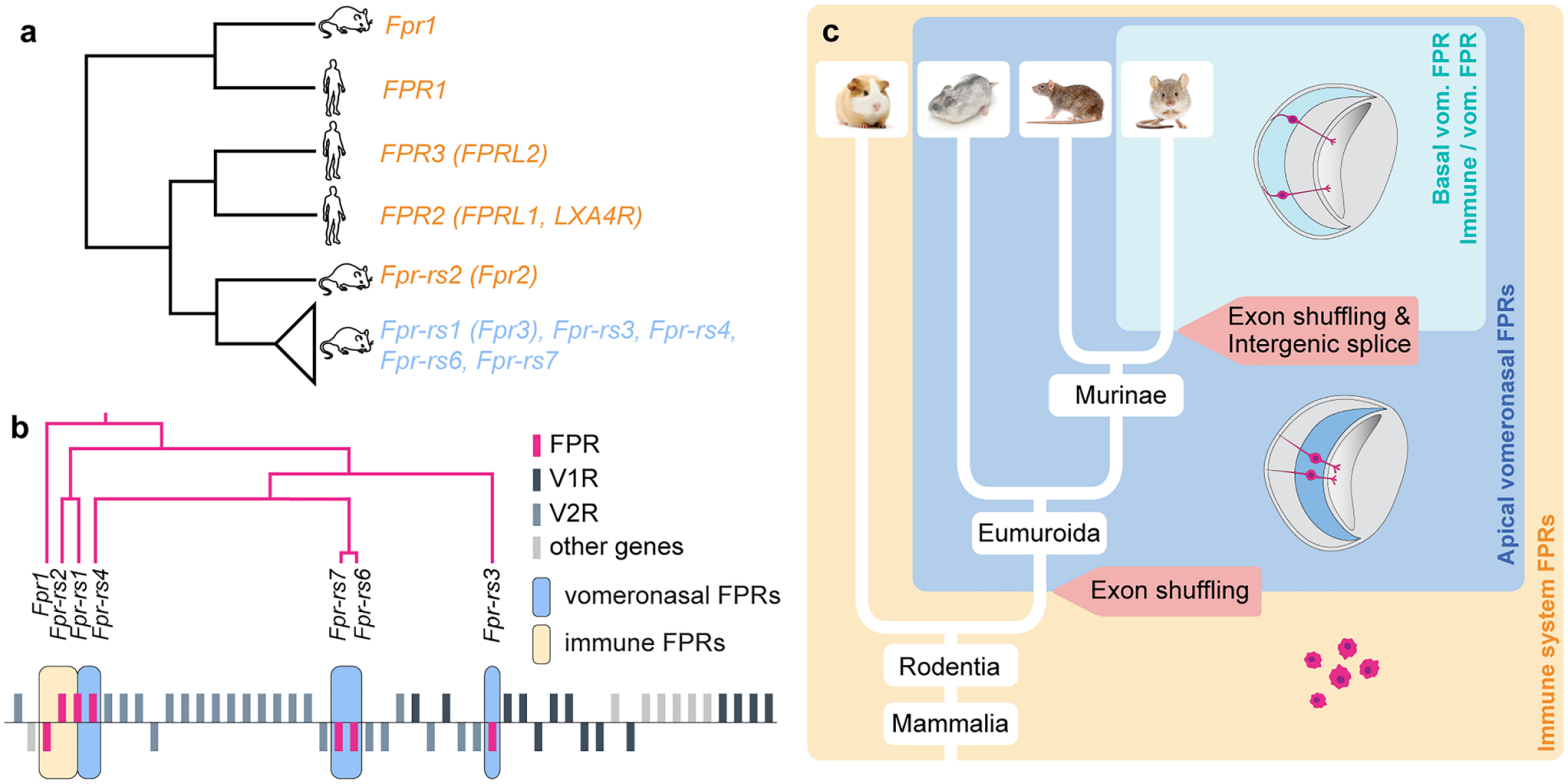
Acquisition of vomeronasal specificity by FPRs. **a** Dendrogram representing the phylogenetic relationships between human and mouse FPR genes. The length of branches does not show distances. **b** Dendrogram showing phylogenetic relationships of mouse FPR genes and their genomic position. Adapted from Dietschi et al. (2017). **c** Schematic indicating the evolution of FPR expression in rodents, showing the timing of apical and basal vomeronasal sensory neuron expression acquisition by FPRs. Extracted from Dietschi et al. (2017)

Most mammalian genomes contain two immune FPR genes, namely in humans *FPR1* and *FPR2* (also called *FPRL1* or *LXA4R*) (Fig. 1a). A large number of primates also benefit from a third FPR gene, termed *FPR3* (also known as *FPRL2*). Phylogenetic analyses of immune mammalian FPR genes revealed that a first duplication led to the divergence of *FPR1* and *FPR2* and that a second duplication of *FPR2* early in primate evolution led to *FPR3* (Dietschi et al. 2017; Liberles et al. 2009; Migeotte et al. 2006; Muto et al. 2015). The ligand specificity of immune FPRs seems to be conserved among species. Predictably, ligands activating specific human FPRs also activate the corresponding mouse FPRs (Bufe et al. 2015), suggestive of a conserved function played by these chemoreceptors in the immune system. Interestingly, the FPR gene family did largely expand in rodents. The mouse genome indeed comprises 7 members: *Fpr1*, which is the mouse ortholog of the human *FPR1*, and *Fpr-rs2*,* Fpr-rs1*,* Fpr-rs3*,* Fpr-rs4*,* Fpr-rs6*, and *Fpr-rs7* (*Fpr-rs2* being the ancestor of the last five, and the ortholog of the ancestor of human *FPR2* and *FPR3*) (Fig. 1b) (Dietschi et al. 2017; Gao et al. 1998; Wang and Ye 2002). Of note, *Fpr-rs1* is often wrongfully considered to represent an ortholog of the primate *FPR3* and has thus been termed *Fpr3* in recent publications and official gene databases (MGI: 1194495, NCBI Gene: 14294). However, *Fpr-rs1* resulted from a duplication of *Fpr-rs2*, which is most closely related to the human *FPR2*. To avoid any confusion, we will here call this gene *Fpr-rs1*.

The functional importance of these additional FPR genes in rodents remained elusive until 2009, when two groups independently discovered that *Fpr-rs1*,* Fpr-rs3*,* Fpr-rs4*,* Fpr-rs6*, and *Fpr-rs7* were almost exclusively expressed in sensory neurons of the vomeronasal organ (Liberles et al. 2009; Riviere et al. 2009). Naturally, this novel tissue specificity suggested a potential neofunctionalization of these FPRs, since they switched from being internal pathogen sensors to olfactory chemoreceptors.

## Expression and evolution of FPRs in the vomeronasal system

The vomeronasal organ is an olfactory sensor specialized in the detection of chemical cues that trigger hardwired, innate behaviors, related to survival and reproduction, including sexual interactions (Ferrero et al. 2013; Haga et al. 2010; Kimchi et al. 2007; Leypold et al. 2002; Stowers and Liberles 2016), aggression and territorial behaviors (Chamero et al. 2007; Kaur et al. 2014), predator avoidance (Papes et al. 2010), parenting (Dulac et al. 2014; Tachikawa et al. 2013), and avoidance of sick conspecifics (Boillat et al. 2015). Two different neuronal populations are present in the vomeronasal organ. The first one is located apically and the second basally in the sensory neuroepithelium. Vomeronasal sensory neurons (VSNs) express vomeronasal type 1 and type 2 receptors (V1Rs and V2Rs respectively), both pertaining to the G-coupled receptor family (Dulac and Axel 1995; Herrada and Dulac 1997; Matsunami and Buck 1997; Ryba and Tirindelli 1995). Apical VSNs express V1Rs together with the G-protein Gα_i2_, and basal VSNs express V2Rs together with Gα_o_. *Fprs-rs3*, *Fprs-rs4*, *Fprs-rs6*, and *Fprs-rs7* are expressed in the apical layer of the vomeronasal sensory epithelium, mimicking the expression of V1R genes, and *Fpr-rs1* is expressed in the basal layer of the vomeronasal sensory epithelium, along with V2R genes (Liberles et al. 2009; Riviere et al. 2009). FPR-expressing neurons are intermingled with V1R/V2R-expressing neurons within the sensory epithelium and constitute about 4% of the total VSN population. Remarkably, vomeronasal FPRs appear to be entirely integrated in the vomeronasal system, fulfilling all the criteria required for functional chemoreception. First, these FPR-expressing neurons co-express either Gα_i2_ or Gα_o_, depending on their apical or basal expression pattern. Second, receptor gene expression follows the singular expression rule that governs V1R gene transcription, that is each sensory neuron expresses a single vomeronasal receptor gene from a single allele. This singular expression rule is fundamental to the circuit logic of the whole olfactory system, as the expressed chemoreceptor determines both the odorant receptive field and the axonal targeting in the brain of each neuron (Vassalli et al. 2002). Indeed, axons of neurons expressing the same receptor gene converge at specific locations within the olfactory bulb, where they coalesce to form glomeruli. This is true for odorant receptors (Mombaerts et al. 1996), V1Rs, V2Rs (Belluscio et al. 1999; Rodriguez et al. 1999), and FPRs (at least for the vomeronasal FPR whose corresponding circuit has been mostly studied, *Fpr-rs3* (Dietschi et al. 2013)). This homophilic coalescence, still poorly understood today, is dependent on the identity of the receptor. Third, the very low K_a_/K_s_ ratio of *Fpr-rs3* indicates that this gene is under purifying selection in rodents (Dietschi et al. 2017). Finally, the fixation of multiple amino acid residues in vomeronasal FPRs that are absent in immune FPRs are indicative of novel characteristics possibly linked to agonist recognition (Dietschi et al. 2017). In summary, FPR-expressing neurons in the vomeronasal organ display the main characteristics of functional olfactory neurons.

How did immune receptors acquire vomeronasal tissue specificity, and how did they integrate into this sensory system? Phylogenetic analyses of FPR genes in mammalian species showed that the split between immune and vomeronasal FPRs occurred at the root of the Eumuroida group (Fig. 1c) (Dietschi et al. 2017), which includes more than 1600 current species and represents the most evolutionarily successful mammalian clade (Steppan et al. 2004). Immune FPRs, which all contain a single coding exon and are grouped together in the genome of mammals, are juxtaposed with a large vomeronasal receptor gene cluster containing both V1R and V2R genes. However, in murid rodents and unlike other mammalian species, a number of FPR genes are embedded inside this VR cluster (Dietschi et al. 2017). These unusually located FPR genes are those expressed in the vomeronasal organ. Comparison of the exon sequences of FPR and V1R genes between multiple species allowed the retracing of the events that led to their vomeronasal acquisition in rodents. First, in a rodent living approximately 70 million years ago (Fang et al. 2014), a gene shuffling event led to the integration of an FPR coding exon (likely the immune *Fpr-rs2*), downstream of a V1R promoter and first exon. This generated a splice trap that hijacked the endogenous V1R regulatory sequences, the pseudogenization of the endogenous V1R coding sequence, and the emergence of *Fpr-rs3*, the first vomeronasal FPR. The cluster of apically expressed FPRs later expanded through several duplications, leading to the birth of *Fpr-rs4*, *Fpr-rs6*, and *Fpr-rs7*. Consistent with this series of events, the promoter region of all apically expressed vomeronasal FPRs is highly similar to that of *Fpr-rs3.*

In contrast, the regulatory sequences of *Fpr-rs1* are very different. Over 50 million years after the first FPR exon shuffling event, after the split between mice and rats, a second shuffling event took place (Fig. 1c). It is thus only observed in the *Mus* genus. This genomic accident led to the integration of a copy of the immune *Fpr-rs2* coding sequence just downstream of the first exon of a V2R gene. Again, this duplicated FPR gene benefited from a VR regulatory sequence, but this time of a V2R. As a result, this novel FPR became transcribed in basal vomeronasal sensory neurons and exhibits, unlike all other vomeronasal FPRs, regulatory sequences homologous to the ones characteristic of V2R genes. In summary, at two separate time points, an FPR coding sequence was integrated in a VR gene cluster, and hijacked VR-specific regulatory elements, leading to their expression in the vomeronasal organ.

Is there something particularly permissive within the VR cluster that allowed this neofunctionalization process of FPRs to happen twice? In fact, all olfactory chemoreceptor families exhibit a high rate of birth and death, which is particularly pronounced in those expressed in the vomeronasal system. Various accidents lead to this rapid evolution, accidents that range from simple duplications of whole chemoreceptor genes, to the generation of more complex chimeras between coding sequences. This instability is likely favorable in terms of survival. Indeed, the variability of the olfactory receptor repertoires is not only different between species but also present across individuals inside a given species. This toolbox diversity may be advantageous to a community in various stress situations. The olfactory system may thus have evolved as an innovation machine that is prone during evolution at expanding or contracting tools of a given type, a versatility that as a side effect may also make it amenable to host foreign GPCRs (that is adopting non olfactory GPCRs such as immune FPRs). Possibly critical for this dynamic reshuffling are the long interspersed nuclear element (LINE) repeats that are enriched within V1R, V2R, and OR gene clusters (Kambere and Lane 2009), since these elements favor unequal recombinations and generally correlate with a high propensity for local duplication events.

## The reverse evolution of Fpr-rs1

**Fig. 2:**
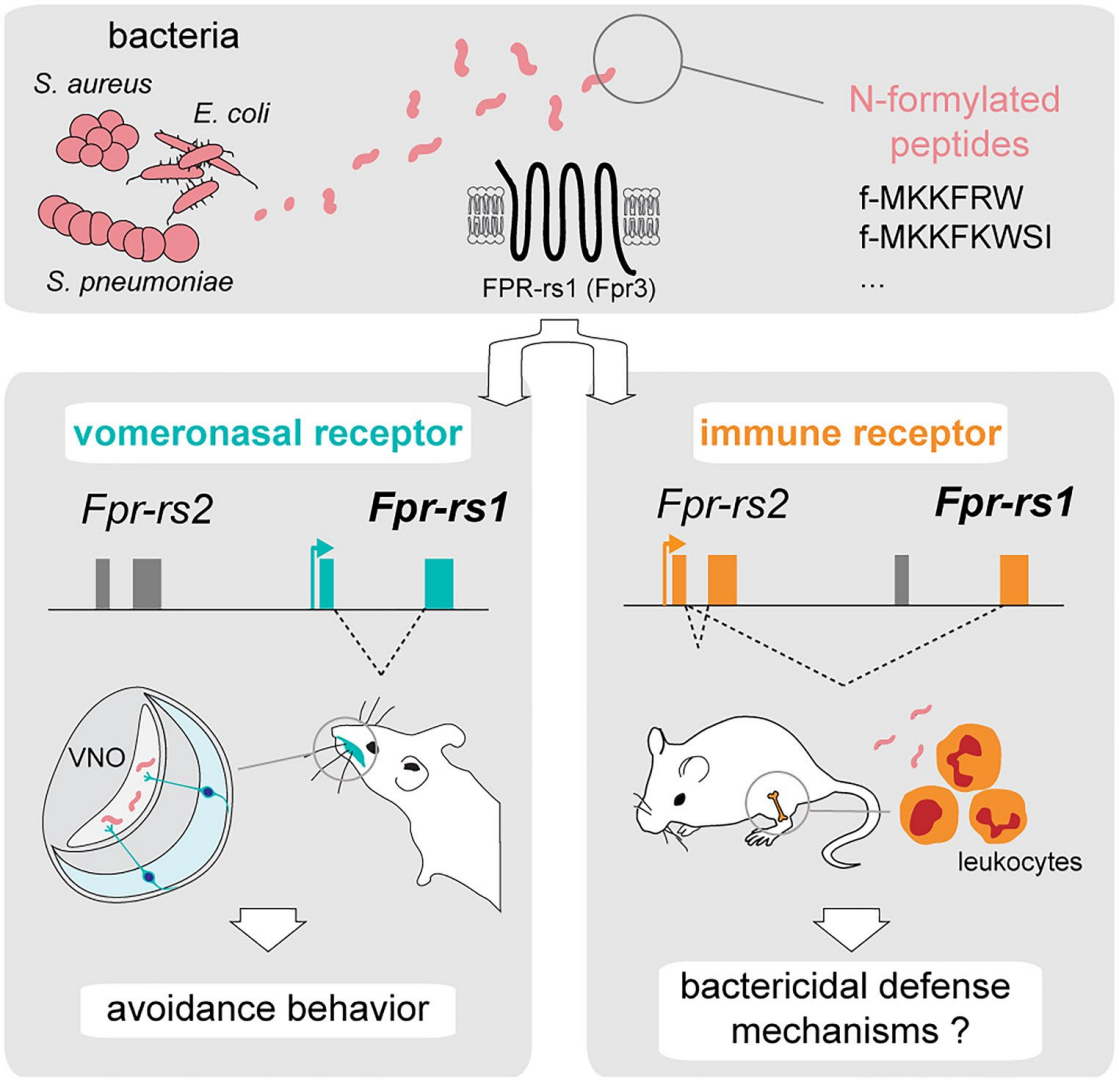
Dual identity of FPR-rs1. Upper panel, formylated peptides from pathogenic bacteria activate FPR-rs1. *Fpr-rs1* can be transcribed in both the vomeronasal organ and the immune system. Left panel, in the vomeronasal organ, *Fpr-rs1* is expressed in the basal layer of the sensory epithelium, driven by a promoter inherited from a V2R gene. Formylated peptides from pathogenic bacteria activate vomeronasal FPR-rs1 and induce avoidance behavior in mice. Right panel, in the immune system, *Fpr-rs1* is expressed in bone marrow cells, where an intergenic splice allows the *Fpr-rs2* promoter to drive its expression. The blue and orange arrows correspond to the vomeronasal *Fpr-rs1* and immune *Frp-rs2/Fpr-rs1* transcription start sites, respectively. FPR-rs1 in leukocytes could play a role in bactericidal defense mechanisms, similarly to other immune FPRs

*Fpr-rs1* is different from other vomeronasal FPR genes. Not only is it expressed in the basal vomeronasal neuroepithelium, but its transcription rate in the immune system can be increased under specific conditions (Dietschi et al. 2017). Indeed, after an inflammatory challenge with lipopolysaccharides (LPS), *Fpr-rs1* expression is upregulated in immune cells, as are the original immune FPR genes (Mandal et al. 2005; Stempel et al. 2016). The analysis of *Fpr-rs1* transcripts from the vomeronasal organ and from immune cells present in bone marrow revealed the presence of two transcript variants (Fig. 2). While in the vomeronasal organ expression is driven by the *Fpr-rs1* promoter, in immune cells, a splice donor site within the *Fpr-rs2* first noncoding exon hijacks the splice acceptor site of the *Fpr-rs1* coding exon, thus generating an intergenic, alternative transcript driven by the immune *Fpr-rs2* promoter. This optional return of a modified neuronal FPR to the original tissue specificity of FPRs is remarkable and represents an extraordinary model of evolutionary innovation.

## Function of vomeronasal FPRs

Much of our knowledge on FPR ligands originates from research on immune FPRs. Hence, after the discovery of FPR transcripts in mouse vomeronasal sensory neurons, the question was to know whether FPR-rs1, FPR-rs3, FPR-rs4, FPR-rs6, and FPR-rs7 share ligand affinities with immune FPRs. Initial in vitro and ex vivo screens for ligands showed that vomeronasal FPRs were responsive to several of the known immune-FPR ligands, including fMLF, CRAMP, lipoxin A4, and uPAR (Riviere et al. 2009). Whether these ligands activate vomeronasal FPRs in vivo at naturally occurring ligand concentrations as well as the functional relevance of this agonistic activity remains to be defined. As mentioned, formylated peptides belong to the most effective immune FPR ligands. The agonist potential of formylated peptides was extensively tested for the mouse FPR-rs1 in a heterologous expression assay, revealing that FPR-rs1 responds to some formylated bacterial signal peptides, but this vomeronasal receptor appears more narrowly tuned than immune FPRs. Recently, it was shown that HEK293T cells expressing FPR-rs1 respond to the formylated peptide motif f-MKKFRW (Bufe et al. 2019), a signal peptide present in membrane proteins of a large family of Gram-negative bacteria, the *Enterobacteriaceae* (which include *Escherichia*, *Salmonella*, and *Shigella*, as well as a few Gram-positive bacteria). More specifically, the MKKFRW motif is highly conserved at the N-terminus of the virulence-regulating factor protein MgrB of these bacteria. Most relevant perhaps was the observation that this peptide triggers the activation of basal vomeronasal sensory neurons expressing *Fpr-rs1.* Moreover, the peptide induces avoidance behavior in male mice when added to female estrus urine, which otherwise is a highly attractive stimulus to males. Mice lacking the FPR-rs1 receptor or components of the vomeronasal signaling cascade do not display any avoidance of the MgrB formylated peptide, clearly suggesting a critical role for FPR-rs1 as a chemoreceptor involved in the behavioral response to bacterial cues (Fig. 2) (Bufe et al. 2019).

Mice have the ability to discriminate between sick and healthy conspecifics (Boillat et al. 2015). Sickness-related cues emitted by mice that are in an acute inflammatory state or infected by a virus trigger the activation of vomeronasal sensory neurons and induce aversive behaviors in the receiver. This behavior is dependent on vomeronasal function (Boillat et al. 2015). To date, the identity of the vomeronasal chemoreceptors mediating sick-conspecific avoidance remains unknown. Because of the affinity of both immune and vomeronasal FPRs for pathogen- and immune-associated ligands, it has been speculated that vomeronasal FPRs might play a role in mediating sick conspecific avoidance behaviors (Boillat et al. 2015; Dietschi et al. 2017). It was shown that mice lacking *Fpr-rs1* are still capable of discriminating between healthy and immune-challenged peers, thus excluding that FPR-rs1 alone is responsible for this behavior (Bufe et al. 2019). Further studies with the deletion of the whole repertoire of vomeronasal FPRs will thus be necessary to define their potential role in the avoidance of sickness-related cues emitted by conspecifics. Less obvious maybe, another possible function of vomeronasal FPRs is the assessment of the microbiota of conspecifics. Over the past 20 years, it has become increasingly clear that the microbiome plays an important role in the development and health of the brain, as well as in central nervous system disorders (Bastiaanssen et al. 2019). Given the importance of the microbiota for general health, to benefit from a chemosensory tool capable of evaluating the identity of gut microorganisms associated with a conspecific might represent a significant advantage. What type of information may be extracted from the microbiome by the vomeronasal organ has not yet been investigated, and certainly merits further research.

## Conclusion

For several decades, FPRs were known for playing a critical role in the regulation of immune functions, responding to chemoattractants linked to pathogens and to inflammatory processes and triggering appropriate cellular responses. In the present review, we described the evolution of FPR genes and in particular the genomic events that led to their expansion and neofunctionalization in rodents, a lineage in which they appear to be involved in the innate behavioral response to pathogenic cues present in the environment. The acquisition of neuronal specificity by FPRs represents thus a remarkable example of genetic innovation, transitioning from pathogen sensors in the immune system to olfactory receptors in neurons.

## References

[CR1] Bastiaanssen TFS, Cowan CSM, Claesson MJ, Dinan TG, Cryan JF (2019). Making sense of … the microbiome in psychiatry. Int J Neuropsychopharmacol.

[CR2] Belluscio L, Koentges G, Axel R, Dulac C (1999). A map of pheromone receptor activation in the mammalian brain. Cell.

[CR3] Boillat M, Challet L, Rossier D, Kan C, Carleton A, Rodriguez I (2015). The vomeronasal system mediates sick conspecific avoidance. Curr Biol.

[CR4] Bufe B, Zufall F (2016). The sensing of bacteria: emerging principles for the detection of signal sequences by formyl peptide receptors. Biomol Concepts.

[CR5] Bufe B, Schumann T, Kappl R, Bogeski I, Kummerow C, Podgorska M, Smola S, Hoth M, Zufall F (2015). Recognition of bacterial signal peptides by mammalian formyl peptide receptors: a new mechanism for sensing pathogens. J Biol Chem.

[CR6] Bufe B, Teuchert Y, Schmid A, Pyrski M, Pérez-Gómez A, Eisenbeis J, Timm T, Ishii T, Lochnit G, Bischoff M (2019). Bacterial MgrB peptide activates chemoreceptor Fpr3 in mouse accessory olfactory system and drives avoidance behaviour. Nat Commun.

[CR7] Carp H (1982). Mitochondrial N-formylmethionyl proteins as chemoattractants for neutrophils. J Exp Med.

[CR8] Chamero P, Marton TF, Logan DW, Flanagan K, Cruz JR, Saghatelian A, Cravatt BF, Stowers L (2007). Identification of protein pheromones that promote aggressive behaviour. Nature.

[CR9] Dalbey RE, Wang P, van Dijl JM (2012). Membrane proteases in the bacterial protein secretion and quality control pathway. Microbiol Mol Biol Rev.

[CR10] Dietschi Q, Assens A, Challet L, Carleton A, Rodriguez I (2013). Convergence of FPR-rs3-expressing neurons in the mouse accessory olfactory bulb. Mol Cell Neurosci.

[CR11] Dietschi Q, Tuberosa J, Rösingh L, Loichot G, Ruedi M, Carleton A, Rodriguez I (2017). Evolution of immune chemoreceptors into sensors of the outside world. Proc Natl Acad Sci.

[CR12] Dulac C, Axel R (1995). A novel family of genes encoding putative pheromone receptors in mammals. Cell.

[CR13] Dulac C, O’Connell LA, Wu Z (2014). Neural control of maternal and paternal behaviors. Science.

[CR14] Fang X, Nevo E, Han L, Levanon EY, Zhao J, Avivi A, Larkin D, Jiang X, Feranchuk S, Zhu Y (2014). Genome-wide adaptive complexes to underground stresses in blind mole rats Spalax.

[CR15] Ferrero DM, Moeller LM, Osakada T, Horio N, Li Q, Roy DS, Cichy A, Spehr M, Touhara K, Liberles SD (2013). A juvenile mouse pheromone inhibits sexual behaviour through the vomeronasal system. Nature.

[CR16] Gao JL, Chen H, Filie JD, Kozak CA, Murphy PM (1998). Differential expansion of the N-formylpeptide receptor gene cluster in human and mouse. Genomics.

[CR17] Haga S, Hattori T, Sato T, Sato K, Matsuda S, Kobayakawa R, Sakano H, Yoshihara Y, Kikusui T, Touhara K (2010). The male mouse pheromone ESP1 enhances female sexual receptive behaviour through a specific vomeronasal receptor. Nature.

[CR18] Herrada G, Dulac C (1997). A novel family of putative pheromone receptors in mammals with a topographically organized and sexually dimorphic distribution. Cell.

[CR19] Kambere MB, Lane RP (2009). Exceptional LINE density at V1R loci: the Lyon repeat hypothesis revisited on autosomes. J Mol Evol.

[CR20] Kaur AW, Ackels T, Kuo TH, Cichy A, Dey S, Hays C, Kateri M, Logan DW, Marton TF, Spehr M (2014). Murine pheromone proteins constitute a context-dependent combinatorial code governing multiple social behaviors. Cell.

[CR21] Kimchi T, Xu J, Dulac C (2007). A functional circuit underlying male sexual behaviour in the female mouse brain. Nature.

[CR22] Leypold BG, Yu CR, Leinders-Zufall T, Kim MM, Zufall F, Axel R (2002). Altered sexual and social behaviors in trp2 mutant mice. Proc Natl Acad Sci U A.

[CR23] Liberles H, LF, Kuang D, Contos JJ, Wilson KL, Siltberg-Liberles J, Liberles DA, Buck LB (2009). Formyl peptide receptors are candidate chemosensory receptors in the vomeronasal organ. Proc Natl Acad Sci.

[CR24] Mandal P, Novotny M, Hamilton TA (2005). Lipopolysaccharide induces formyl peptide receptor 1 gene expression in macrophages and neutrophils via transcriptional and posttranscriptional mechanisms. J Immunol.

[CR25] Matsunami H, Buck LB (1997). A multigene family encoding a diverse array of putative pheromone receptors in mammals. Cell.

[CR26] Migeotte I, Communi D, Parmentier M (2006). Formyl peptide receptors: a promiscuous subfamily of G protein-coupled receptors controlling immune responses. Cytokine Growth Factor Rev.

[CR27] Mombaerts WF, Dulac C, Chao SK, Nemes A, Mendelsohn M, Edmondson J, Axel R (1996) Visualizing an olfactory sensory map. Cell 87:675–686 10.1016/s0092-8674(00)81387-2 8929536

[CR28] Muto Y, Guindon S, Umemura T, Kőhidai L, Ueda H (2015). Adaptive evolution of formyl peptide receptors in mammals. J Mol Evol.

[CR29] Papes F, Logan DW, Stowers L (2010). The vomeronasal organ mediates interspecies defensive behaviors through detection of protein pheromone homologs. Cell.

[CR30] Rabiet MJ, Huet E, Boulay F (2005). Human mitochondria-derived N-formylated peptides are novel agonists equally active on FPR and FPRL1, while Listeria monocytogenes-derived peptides preferentially activate FPR. Eur J Immunol.

[CR31] Riviere S, Challet L, Fluegge D, Spehr M, Rodriguez I (2009). Formyl peptide receptor-like proteins are a novel family of vomeronasal chemosensors. Nature.

[CR32] Rodriguez I, Feinstein P, Mombaerts P (1999). Variable patterns of axonal projections of sensory neurons in the mouse vomeronasal system. Cell.

[CR33] Ryba NJ, Tirindelli R (1995). A novel GTP-binding protein gamma-subunit, G gamma 8, is expressed during neurogenesis in the olfactory and vomeronasal neuroepithelia. J Biol Chem.

[CR34] Schiffmann E, Corcoran BA, Wahl SM (1975). N-formylmethionyl peptides as chemoattractants for leucocytes. Proc Natl Acad Sci.

[CR35] Stempel H, Jung M, Pérez-Gómez A, Leinders-Zufall T, Zufall F, Bufe B (2016). Strain-specific loss of formyl peptide receptor 3 in the murine vomeronasal and immune systems. J Biol Chem.

[CR36] Steppan SJ, Adkins RM, Anderson J (2004). Phylogeny and divergence-date estimates of rapid radiations in muroid rodents based on multiple nuclear genes. Syst Biol.

[CR37] Stowers L, Liberles SD (2016). State-dependent responses to sex pheromones in mouse. Curr Opin Neurobiol.

[CR38] Tachikawa KS, Yoshihara Y, Kuroda KO (2013). Behavioral transition from attack to parenting in male mice: a crucial role of the vomeronasal system. J Neurosci.

[CR39] Vassalli A, Rothman A, Feinstein P, Zapotocky M, Mombaerts P (2002). Minigenes impart odorant receptor-specific axon guidance in the olfactory bulb. Neuron.

[CR40] Wang ZG, Ye RD (2002). Characterization of two new members of the formyl peptide receptor gene family from 129S6 mice. Gene.

[CR41] Weiß E, Kretschmer D (2018). Formyl-peptide receptors in infection, inflammation, and cancer. Trends Immunol.

[CR42] Ye RD, Boulay F, Wang JM, Dahlgren C, Gerard C, Parmentier M, Serhan CN, Murphy PM (2009). International Union of Basic and Clinical Pharmacology. LXXIII. Nomenclature for the formyl peptide receptor (FPR) family. Pharmacol Rev.

